# Physical Therapy Considerations for Chronic Kidney Disease and Secondary Sarcopenia

**DOI:** 10.3390/jfmk3010005

**Published:** 2018-01-05

**Authors:** Haniel J. Hernandez, Gideon Obamwonyi, Michael O. Harris-Love

**Affiliations:** 1 Muscle Morphology, Mechanics and Performance Laboratory, Human Performance Research Unit, Clinical Research Center, Veterans Affairs Medical Center, Washington, DC 20422, USA; 2 Physical Medicine & Rehabilitation Service, Veterans Affairs Medical Center, Washington, DC 20422, USA; 3 Department of Exercise and Nutritional Sciences, Milken Institute School of Public Health, The George Washington University, Washington, DC 20052, USA; 4 Geriatrics and Extended Care Service/Research Service, Veterans Affairs Medical Center, Washington, DC 20422, USA

**Keywords:** chronic kidney disease, sarcopenia, physical therapy, geriatrics, muscle

## Abstract

Chronic kidney disease (CKD) is a progressive condition that may negatively affect musculoskeletal health. These comorbidities may include malnutrition, osteoporosis, and decreased lean body mass. Secondary sarcopenia due to CKD may be associated with mobility limitations and elevated fall risk. Physical therapists are well-positioned among the health care team to screen for secondary sarcopenia in those with CKD and for the treatment of musculoskeletal comorbid conditions that may affect functional performance. Given the consequences of both low muscle mass and low bone mineral density, appropriate and timely physical therapy is important for fall risk assessment and intervention to minimize the susceptibility to bone fracture. While strength training has been studied less frequently than aerobic training for the management of secondary CKD conditions, evidence suggests that this patient population benefits from participation in strength training programs. However, the provision of a formal exercise prescription by a health care professional, along with formal implementation of an exercise program, may need to be more fully integrated into the standard plan of care for individuals with CKD.

## 1. Introduction

Chronic kidney disease (CKD) is a major health issue that affects millions of adults. A recent account by the Centers for Disease Control and Prevention reveals that 10% of adults living in the U.S. have CKD, which is approximately 20 million people [1,2]. Well-known health conditions secondary to CKD are high blood pressure, malnutrition, bone disorders, and heart and vessel disease. However, lesser known conditions associated with CKD may still have a significant impact on physical functioning and health-related quality of life [3]. Protein-energy wasting syndrome (i.e., “muscle wasting”) may be an under-appreciated condition that has major implications for people with CKD, and merits the attention of physical therapists that provide care for this patient population. The purpose of this report is to summarize the impact of CKD on musculoskeletal health and highlight the role of physical therapy in addressing secondary impairments due to this condition. Understanding the comorbidities associated with kidney disease may aid the rehabilitation approach for individuals with CKD.

## 2. Chronic Kidney Disease and Bone Mineral Density

The main function of the kidney is to regulate water fluid levels within the body, maintain blood pH homeostasis, and remove metabolic byproducts from the blood. In addition, the kidneys play a vital role in hormone production and the reabsorption of glucose and amino acids. CKD causes the kidneys to progressively lose their function over time. The criterion for this disorder is based on a glomerular filtration rate (GFR) of less than 60 mL/min/1.73 m^2^ for more than three months, independent of the cause [2].There are five stages in which CKD is marked by increased severity, with the culminating stage being end-stage renal disease (ESRD). The definition for each stage is depicted in Table 1.

CKD is frequently associated with malnutrition and adverse changes to body composition and musculoskeletal health [4]. For example, diminished bone strength is among the common sequelae that may contribute to disablement. Medical imaging using dual-energy X-ray absorptiometry (DXA) or peripheral computerized tomography (CT) can provide estimates of bone mineral density (BMD) levels and the likelihood of fracture in those with CKD [5]. The risk of “low-energy” fracture is increased two-fold in people with kidney damage [5]. The increased fracture risk for people with CKD is reflected in the disordered bone mineralization, turnover, and linear growth secondary to abnormalities in vitamin D metabolism and serum calcium, phosphate, and parathyroid hormone (PTH) levels [6]. Elevated levels of PTH promote increased osteoclastic bone resorption and diminished levels of vitamin D result in poor bone mineralization, which are worsened by metabolic acidosis. Importantly, vitamin D receptor abnormalities are linked with impaired calcium metabolism [7]. The disordered binding of the hormonally active metabolite of vitamin D to its nuclear receptor affects the messenger RNA gene transcription, resulting in lower muscle protein synthesis. In addition, the interaction between vitamin D and serum calcium may also adversely impact muscle function in people with CKD [8]. Vitamin D deficiency lies at the intersection of osteoporosis and muscle wasting.

## 3. Chronic Kidney Disease and Lean Body Mass

The health complications that accompany CKD result in decreased muscle performance and physical functioning [9]. The loss of lean muscle mass (LBM) secondary to kidney disease is often termed “muscle wasting” since its etiology is largely independent of age with a clinical presentation that appears to be an accelerated form of sarcopenia [10]. Conditions such as CKD, malnutrition, and other chronic diseases are considered causes of secondary sarcopenia [11]. It is important to note that secondary sarcopenia associated with CKD may affect even those in the early stages of the disease [12]. Avin et al. [4] have stated that secondary sarcopenia due to CKD is essentially the result of an altered balance between catabolic and anabolic processes to control muscle homeostasis [4]. Controlling muscular homeostasis is a complex process dependent on hormonal and immunologic factors, as well as progenitor cell function. However, homeostatic balance may be adversely affected by excess inflammation, metabolic acidosis, malnutrition, and physical inactivity. Additionally, muscular degradation results from elevated angiotensin II levels, resistance to insulin-like growth factor 1, and increased myostatin levels, which are all potential consequences of CKD. Conversely, the rate of muscular regeneration is limited due to abnormal myogenic regulatory factors, increased myostatin, mitochondrial dysfunction, and of course, decreased physical activity [4]. It has also been found that type II muscle fiber atrophy is most prevalent in those with CKD who are undergoing dialysis treatment. Collectively, these pathological changes could partially explain the frequent reports of decreased muscle capacity and excessive fatigability in this patient population [13].

Many methods of diagnosing and assessing sarcopenia have been proposed, and these approaches should be considered for the case management of people with CKD and muscle dysfunction. Typically, confirmatory body composition testing results from a positive screening finding (traditionally a habitual gait speed test, with cut off values ranging from 0.8 to 1.0 m/s) during the course of the physical examination and patient history process [14]. Magnetic resonance imaging (MRI) and CT scans have been found to be valid tools to estimate LBM and aspects of muscle quality, but these imaging tools are very costly to use in a clinical setting for serial measurement [15]. Another method of assessing LBM, bioelectrical impedance analysis (BIA), has been proposed as a practical alternative to imaging modalities in multiple clinical settings [16]. DXA remains the method most often used to assess LBM [17] and is often expressed as appendicular lean mass (aLM/ht^2^). However, the disadvantage of utilizing DXA is its higher cost and low-level radiation exposure in comparison to BIA.

While DXA is the preferred method for hospital settings and is more reliable than BIA, it must be noted that BIA is a lower-cost option and is more readily available in outpatient and community health settings. However, important limitations exist regarding body composition estimates using BIA for those with altered states of hydration. Estimates of LBM include extracellular water and BIA may overestimate hydration levels and LBM prior to hemodialysis in patients with end-stage renal disease [18]. While both multi-frequency and single-frequency BIA may be used to estimate LBM in people with renal dysfunction, multi-frequency BIA with segmental analysis may be preferred when making distinctions among total body water, extracellular water, and body cell mass [19,20]. Estimates of LBM or body water from a single session should be used with caution in people with altered states of hydration, especially in those transitioning from dehydration to euvolemia [20]. Serial BIA measures before and after hemodialysis may help to guide an interdisciplinary plan of care for people with end-stage renal disease. Careful attention to hydration status and food intake, activity levels, time of day, and other confounding factors are also important to note prior to serial BIA measurement sessions. Importantly, appropriately trained physical therapists and other health care professionals can conduct a body composition assessment with BIA, or alternate methods such as quantitative diagnostic ultrasound [21,22] during the course of the initial clinical referral and visit. Increasingly, it is recognized that muscle mass measures alone do not fully encompass the important clinical markers of muscle health [22,23]. Standardized measures of both muscle strength and functional performance are now integral elements of the sarcopenia assessment [24], which lend value to the skills provided by physical therapists and their role on the health care team.

## 4. The Clinical Management of Secondary Sarcopenia Associated with Chronic Kidney Disease

**Figure 1:**
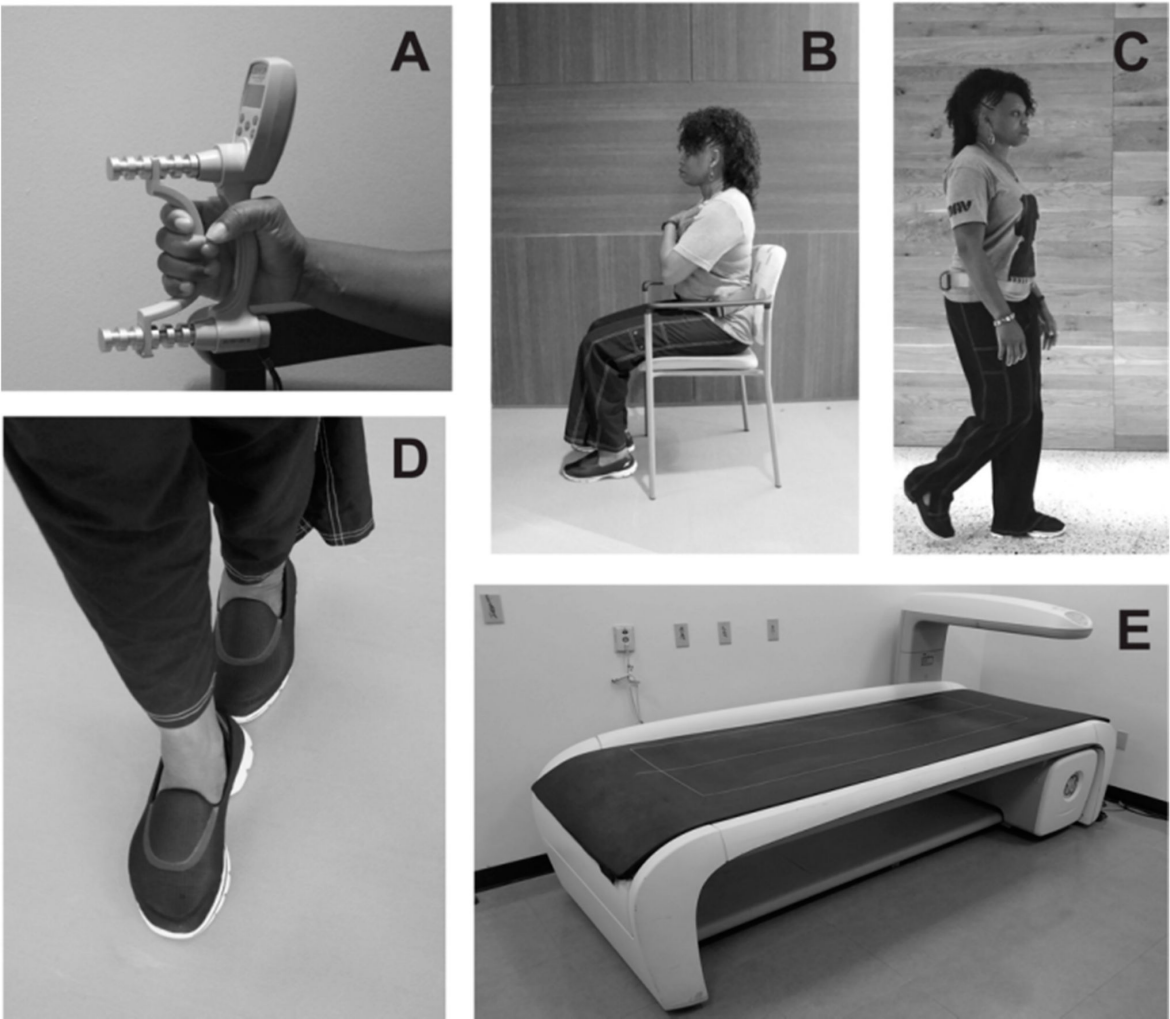
Sarcopenia Screening and Staging Outcomes. Sarcopenia screening and staging criteria include measures of muscle mass, strength, and function. Grip strength is recognized is an objective impairment measure used for staging, but it has also been proposed as an alternate screening tool (Panel (**A**)). In addition, common functional assessments for sarcopenia include the repeated sit-to-stand test and habitual gait speed (Panel (**B–C**)). While no single measure has been designated to characterize functional status for sarcopenia staging, an outcome used with increasing frequency is the Short Physical Performance Battery (SPPB). The SPPB includes commonly used physical assessments such as gait speed (4 m), static balance testing, and the repeated sit-to-stand test (Panels (**B–D**)). Detection of functional limitations or low grip strength may merit confirmatory standardized assessments of muscle mass. Dual-energy X-ray absorptiometry (Panel (**E**)) is often used to estimate muscle mass in medical centers, but alternative assessment methods such as bioelectrical impedance analysis may be used in ambulatory clinics and other settings.

The classification and staging of sarcopenia [24] includes not only the loss of muscle mass, but also the impact of poor body composition on muscle strength and mobility status (Figure 1). Therefore, the physical therapy assessment must include these domains during the examination and evaluation process. Consensus-based criteria for “sarcopenia” includes low muscle mass with either low muscle strength or limited function, whereas the criteria for “severe sarcopenia” include diminished muscle mass, strength, and function [14,24]. Consequently, hand grip dynamometry along with standardized physical performance-based testing can be used as assessment measures for sarcopenia staging. These assessment tools are appropriate for documenting and monitoring changes in physical performance as a result of sarcopenia, regardless of the cause [17]. However, physical therapists may want to expand the quantitative strength assessment beyond simple grip dynamometry for individuals with mobility limitations that involve the lower extremities [25]. Moreover, comorbid factors such as diminished BMD and muscle strength require the assessment of fall risk in those with CKD. Tools ranging from the performance-based Timed Up-and-Go test to questionnaires that assess fall avoidance behavior such as the Falls Efficacy Scale help to comprise a comprehensive assessment approach [23]. In addition, the Short Physical Performance Battery, a commonly used outcome in sarcopenia studies, features static balance testing as individual test items [26]. Finally, emerging tools for the detection and management of sarcopenia include the Sarcopenia and Quality of Life questionnaire (SarQoL), a newly developed quality of life scale specifically designed for those with sarcopenia [27] and a brief five-item screening questionnaire (SARC-F) with items related to strength, function, and accidental falls [28].

Although sarcopenia is fairly new to the public as a formal diagnosis (ICD-10-CM code: M62.84), it has been a topic of interest by clinicians and investigators for decades [29]. There have been various approaches suggested to combat sarcopenia and minimize its impact. Among these suggestions are dietary modifications and increased exercise to promote cardiopulmonary and muscular fitness. Anaerobic exercise has been shown to slow down the rate of muscle mass and strength decline with advancing age [30]. Even aerobic exercise may improve the cross-sectional area (CSA) of muscular fibers in some untrained individuals, despite the use of this exercise mode to improve endurance capacity and cardiopulmonary fitness rather than muscle hypertrophy [30]. There have been many studies published on the effects of varying anaerobic exercise regimens on older adults and how it has improved their strength levels and muscle mass [31,32]. For example, investigators have demonstrated improvements in muscular CSA of 11%, along with improvements exceeding 100% for muscular strength, following a simple high-intensity 12-week training regimen in older men [33]. In addition, many investigators have come to the conclusion that managing sarcopenia with combinations of progressive strength training, appropriate supplementation of vitamin D, and monitored protein intake may lead to improvements in fall and fracture risk in those with CKD-related sarcopenia [34,35]. Other study findings suggest that progressive strength training alone has been effective in improving muscle mass in patients with late stage CKD (stage 3b to 4). The progressive strengthening regimen conducted by Watson et al. [36] included sessions that occurred three times per week over the course of eight weeks. Not only was body weight improved, but muscle volume and exercise capacity demonstrated increases as well [36]. The primary strengthening exercise was limited to knee extension, but it did demonstrate local anabolic effects at the target muscle group, which also lead to improvements in functional performance. Notably, the authors of this study mentioned the potential advantages of a home or community-based approach to training in comparison to clinic-based intervention programs.

Physical inactivity and a lack of regular exercise remain as key modifiable risk factors for general morbidity. Moreover, the need for health care practitioners to provide a formal exercise prescription to people with chronic disease has been recognized by multiple stakeholders [37,38]. Strength training confers benefits to multiple patient populations, but it also may specifically address contributing factors to sarcopenia such as sedentary behavior, decreased muscle mass, reduced neural stimulation, and lipotoxicity [39]. While exercise has been shown to be safe and effective for people with CKD, optimal programming, implementation strategies, and long-term effectiveness associated with strengthening regimens have been studied less in comparison to aerobic training regimens for this patient population [35]. The progression of exercise intensity from moderate to vigorous (3.5–7.0 to 7.0 kcal/min) is acceptable for the exercise programming for many people with chronic disease, and may be considered for those with kidney dysfunction [5,40]. The implementation of formalized strengthening programs for people with CKD requires appropriate attention to cardiovascular risk factors and awareness of exercise-related hypertension [41]. Modifications to the exercise prescription based on blood pressure control and nutritional status may need to be made for individuals with CKD. Moreover, the exercise prescription must be tailored specifically to the needs and ability of the individual within the context of any existing comorbidities and the prior level of activity. Supplementary approaches to combat sarcopenia include dietary modifications and improving quality protein intake. However, nutritional consultation among the health care team may be needed to regulate protein intake in order to avoid dietary complications such as proteinuria in people with CKD.

## 5. Discussion

The sequalae associated with CKD such as secondary sarcopenia due to protein-energy wasting syndrome and compromised bone strength requires a focused rehabilitation assessment and plan of care that includes:

- Sarcopenia screening with confirmatory assessments of objective muscle strength, functional status, and body composition, when appropriate
- The detection of elevated fall risk along with an assessment of fall avoidance behavior
- A formal exercise prescription designed to increase muscle strength, promote bone health, and improve balance.

Case management of those with CKD will involve an interdisciplinary care team that includes renal care along with physical medicine, radiology, and nutritional services. Physical therapists can play a key role in the timely detection of physical impairments or mobility limitations in people with kidney disorders. In addition, the patient history and other forms of self-report may reveal decreased community participation or the increased need of caregiver assistance due to secondary sarcopenia. The published evidence supports the use of progressive resistive exercise to improve the physical functioning of those with CKD. Given the chronicity of this disorder, physical therapists may work with the care team to monitor changes in muscle performance and fall risk, and also ensure that the exercise prescription remains congruent with the changing abilities and disease status of the patient. Potential areas of future rehabilitation research include examining the relative contributions of muscle mass and muscle strength to disablement in people with CKD, and further examination into the utility of muscle quality assessments to aid the risk stratification for poor health outcomes in those with impaired renal function.

## 6. Conclusions

CKD is a progressive condition that adversely affects musculoskeletal health. Secondary sarcopenia due to CKD is associated with malnutrition, osteoporosis, mobility limitations, and elevated fall risk. Physical therapy constitutes an important element of the plan of care for individuals with CKD. Screening and treating secondary sarcopenia in this patient population may have value given the elevated risk of low-energy fractures and other disabling conditions. Strength training remains an important part of the comprehensive management of CKD. However, greater involvement from the health care team is needed to provide a formal exercise prescription and monitor program effectiveness.

## Figures and Tables

**Table 1:** Stages and classes of chronic kidney disease.

Stage	GFR Level (mL/min/1.73 m^2^)	Description
1	90 or above	Kidney damage that includes normal or high GFR
2	60–89	Kidney damage that includes slightly decreased GFR
3A	45–59	Moderate CKD with mild-moderate decrease in GFR
3B	30–44	Moderate CKD with moderate-severe decrease in GFR
4	15–29	Severe CKD with severe decrease in GFR
5	<15	End stage renal disease/kidney failure where dialysis in required

GFR, glomerular filtration rate; CKD, chronic kidney disease.
